# Combined Inflammatory and Metabolic Defects Reflected by Reduced Serum Protein Levels in Patients with Buruli Ulcer Disease

**DOI:** 10.1371/journal.pntd.0002786

**Published:** 2014-04-10

**Authors:** Richard O. Phillips, Fred S. Sarfo, Jordi Landier, Reid Oldenburg, Michael Frimpong, Mark Wansbrough-Jones, Kabiru Abass, William Thompson, Mark Forson, Arnaud Fontanet, Fatoumata Niang, Caroline Demangel

**Affiliations:** 1 Komfo Anokye Teaching Hospital, Kumasi, Ghana; 2 Kwame Nkrumah University of Science and Technology, Kumasi, Ghana; 3 Institut Pasteur, Unité de Recherche et d'Expertise Epidémiologie des Maladies Emergentes, Paris, France; 4 Institut Pasteur, Unité d'Immunobiologie de l'Infection, Paris, France; 5 CNRS URA 1961, Paris, France; 6 Kumasi Centre for Collaborative Research, Kumasi, Ghana; 7 St George's University of London, London, United Kingdom; 8 Agogo Presbyterian Hospital, Agogo, Ghana; 9 Tepa Government Hospital, Tepa, Ghana; 10 Conservatoire National des Arts et Métiers, Paris, France; Swiss Tropical and Public Health Institute, Switzerland

## Abstract

Buruli ulcer is a skin disease caused by *Mycobacterium ulcerans* that is spreading in tropical countries, with major public health and economic implications in West Africa. Multi-analyte profiling of serum proteins in patients and endemic controls revealed that Buruli ulcer disease down-regulates the circulating levels of a large array of inflammatory mediators, without impacting on the leukocyte composition of peripheral blood. Notably, several proteins contributing to acute phase reaction, lipid metabolism, coagulation and tissue remodelling were also impacted. Their down-regulation was selective and persisted after the elimination of bacteria with antibiotic therapy. It involved proteins with various functions and origins, suggesting that *M. ulcerans* infection causes global and chronic defects in the host's protein metabolism. Accordingly, patients had reduced levels of total serum proteins and blood urea, in the absence of signs of malnutrition, or functional failure of liver or kidney. Interestingly, slow healers had deeper metabolic and coagulation defects at the start of antibiotic therapy. In addition to providing novel insight into Buruli ulcer pathogenesis, our study therefore identifies a unique proteomic signature for this disease.

## Author Summary

Buruli ulcer is a skin disease caused by inoculation of *Mycobacterium ulcerans* bacteria, which is emerging in tropical countries. Lesions typically start as nodules, which eventually open to form progressive, non-healing wounds. Although anti-mycobacterial antibiotics are efficient at stopping the infection, ulcers often persist after completion of treatment for reasons that remain unclear. In the present study, we performed a quantitative analysis of a large array of proteins circulating in peripheral blood, with the view to identifying biomarkers of disease progression and wound chronicity. We found that Buruli ulcer disease induces a unique signature of serum protein suppression that outlasts the elimination of bacteria. Interestingly, the initial levels of several of these proteins differed between fast and slow healers, suggesting an association with healing defects. Our results unravel an unexpected feature of Buruli ulcer disease and show that *M. ulcerans* infection has global and durable effects on the host's protein metabolism.

## Introduction

Buruli ulcer disease (BUD) is a progressive ulceration of the skin that results from infection with *Mycobacterium ulcerans* [1], [2], [3]. BUD is associated with swamps, marshes and wetlands, with the most affected countries in Western Africa. The disease classically starts as a nodule, papule, plaque, or oedema that eventually breaks down to form a painless, necrotic ulcer [4]. In the remote rural areas where the disease is endemic, presentation and diagnosis is often delayed and patients present with large ulcers, which cause significant disability [5]. The recommended treatment for BUD consists of streptomycin and rifampicin daily for eight weeks, with grafting of larger lesions [6], [7], [8]. Although this is effective at killing bacteria [9], 2–10% of individuals develop enlargement of lesions upon treatment [10]. Skin wounds typically heal by progressing through four consecutive phases including haemostasis, inflammation, a proliferative stage with synthesis of extracellular matrix (ECM) then a remodelling of the ECM. Whether and how infection with *M. ulcerans* interferes with this healing process is largely unknown [11]. To improve the current disease management practices and develop personalized treatment plans, it is crucial to better understand the pathophysiology of BUD and identify biological correlates of healing.

*M. ulcerans* produces mycolactone, a macrolide cytotoxin with immunosuppressive properties that underpins bacterial virulence [12]. Sub-cutaneous injection of mycolactone in animal models causes ulcerative skin lesions similar to those seen in BUD [13]. Contrary to wild-type *M. ulcerans*, mycolactone-deficient mutants do not induce defective systemic production of IL-2 in animal models, suggesting that mycolactone also impairs the generation of cellular responses. In line with this hypothesis, mycolactone was shown to suppress the expression of homing receptors and the production of inflammatory mediators by lymphocytes, monocytes and dendritic cells *in vitro* [14], [15], [16], [17], [18], [19]. The mechanism by which mycolactone modulates protein expression in a gene and cell-specific manner in these immune cells nevertheless remains unclear. The action of mycolactone differs from those of known immunosuppressants, acting independently of the mammalian target of rapamycin (mTOR) [14], [19]. Whether mycolactone also alters protein expression in non-immune cells is not documented.

We reported previously that BU patients display a distinctive profile of immune suppression, marked by the down-modulation of selected chemokines and an impaired capacity of T cells to produce cytokines upon stimulation *ex vivo* [20]. In the present study, we performed a larger-scale profiling of serum proteins and standard clinical analyses to improve our global understanding of the effects of *M. ulcerans* infection on the physiology of the host. Our results validate our previous observation that *M. ulcerans* infection down-regulates the serum level of a large array of proteins participating in immune responses. In addition, they show that BUD represses the circulating level of key mediators of coagulation and tissue remodelling. Although these alterations do not impact on the host's vital functions and immunity, they may interfere with wound healing.

## Materials and Methods

### Ethics statement

The protocol of this study was approved by the ethics review committees at the School of Medical Sciences, Kwame Nkrumah University of Science and Technology, Kumasi, Ghana (CHRPE/11/28/06). All adult subjects provided informed consent, and a parent or guardian of any child participant provided informed consent on their behalf. Informed consent was written (with thumb print of parent or guardian in the case of children, depending on literacy). Institutional review board gave approval to document informed consent using thumb print for illiterate participants.

### Study design

Patients were recruited in the middle forest belt of Ashanti Region of Ghana, from Buruli ulcer endemic villages near Tepa Government Hospital (Ahafo Ano North District), Agogo Presbyterian Hospital and Ananekrom Health centre (Asante Akim North District) and Nkawie Government Hospital (Atwima Nwabiagya district), within 70 km of the regional capital Kumasi. Patients were included in the study if they met the WHO clinical case definition of *M. ulcerans* disease; were not pregnant; were not receiving antibiotic treatment; had no history of tuberculosis, leprosy, or liver, kidney, or hearing impairment. Age- and gender-matched healthy controls from the same endemic area were included. Three cohorts of patients and controls were constituted for the purposes of this study that are described in Tables 1 to 4 and Table S1 in Text S1. Body mass index (BMI) was determined in individuals of Cohort 2 according to international classification (WHO global database, http://apps.who.int/bmi/).

**Table 1:** Multi-analyte profiling (Cohort 1).

	Healthy controls (n = 14)	Patients with BUD (n = 20)	
		“Fast healers” (n = 10)	“Slow healers” (n = 10)
Age, median (range), years	15 (7–35)	32 (13–55)	10 (5–35)
Sex, no. male/no. female	7/7	4/6	6/4
Time to healing, median (range), weeks	-	7 (4–12)	29 (16–48)
Ulcer category			
I (lesion size <5 cm in widest diameter)		6	6
II (lesion size <15 cm in widest diameter)		4	4
Age, median (range), years		17 (5–55)	
Time to healing, median (range), weeks		14 (4–48)	

**Table 2:** Complete blood count and blood chemistry (Cohort 2).

Complete blood count and blood chemistry (liver and renal function tests, blood lipids)	Healthy controls (n = 20)	Patients with BUD (n = 44)
Age, median (range), years	14.5 (8–50)	16 (6–61)
Sex, no. male/no. female	7/13	18/26

**Table 3:** FACS analysis (Part of Cohort 2).

	Healthy controls (n = 9)	Patients with BUD (n = 10)
Age, median (range), years	13 (8–35)	25 (12–47)
Sex, no. male/no. female	3/6	3/7
Ulcer category		
I (lesion size <5 cm in widest diameter)		9
II (lesion size <15 cm in widest diameter)		1

**Table 4:** NEFA assay (Cohort 3).

	Healthy controls (n = 9)	Patients with BUD (n = 15)
Age, median (range), years	10 (7–35)	12 (5–32)
Sex, no. male/no. female	5/4	8/7
Time to healing, median (range), weeks	-	18 (4–40)
Ulcer category		
I (lesion size <5 cm in widest diameter)		11
II (lesion size <15 cm in widest diameter)		3

### Diagnosis and treatment

Fine needle aspirates were taken to confirm the clinical diagnosis by PCR for the *IS2404* repeat sequence characteristic of *M. ulcerans*. Patients were started on streptomycin 15 mg/kg and rifampicin 10 mg/kg treatment daily for 8 weeks, as recommended by the WHO, at village health posts under direct observation. Punch biopsy specimens of 4 mm diameter were also stained for acid-fast bacilli and cultured on Lowenstein-Jensen slopes, as previously described [21]. Patients with confirmed BUD of less than 10 cm in maximum diameter provided 2 ml of serum. All lesions had healed by 6 months and there were no recurrences after 12 months upon completion of antibiotic therapy. Healthy contacts from the same endemic areas also provided serum samples to serve as a comparator.

### FACS analysis

Whole blood was collected in heparin-containing tubes and processed within the same day. Mix of fluorochrome-conjugated antibodies were added to 100 µl whole blood, incubated for 15 min in the dark, followed by 15 min in-dark red cell lysis with 1-step Fix/Lyse solution (eBioscience). Cells were washed according to the manufacturer's protocol and analyzed on a BD FACSCalibur. Anti-CD4-FITC (555339), anti-CD8-FITC (345772), anti-CD19-FITC (345788), anti-CD62L-PE (555544), anti-CD45RA-PE (555489) and anti-CD3-APC (555335) were from BD Pharmingen.

### Blood cell counts and chemistry tests

Absolute counts of neutrophils, monocytes, lymphocytes and eosinophils in fresh peripheral blood were determined with a hematology analyzer. Blood glucose, blood lipids (total cholesterol, triglyceride, high and low density lipoprotein HDL and LDL); liver function indicators (Alanine aminotransferase (ALT), alkaline phosphatase (ALP), Gamma-glutamyl transferases (GGT), Total protein, Albumin, Globulin, total/direct/indirect Bilirubin) and renal function indicators (urea, creatinine, blood urea nitrogen to creatinine ratio) were measured in serum with standard hospital instrumentation. Individuals fasted for a minimum of 12 h before LDL, triglyceride or blood glucose measurements. Non-esterified fatty acids (NEFA) were assessed in serum with the Free fatty acids, half-micro test (Roche).

### Multi-analyte profiling of serum proteins

**Figure 1:**
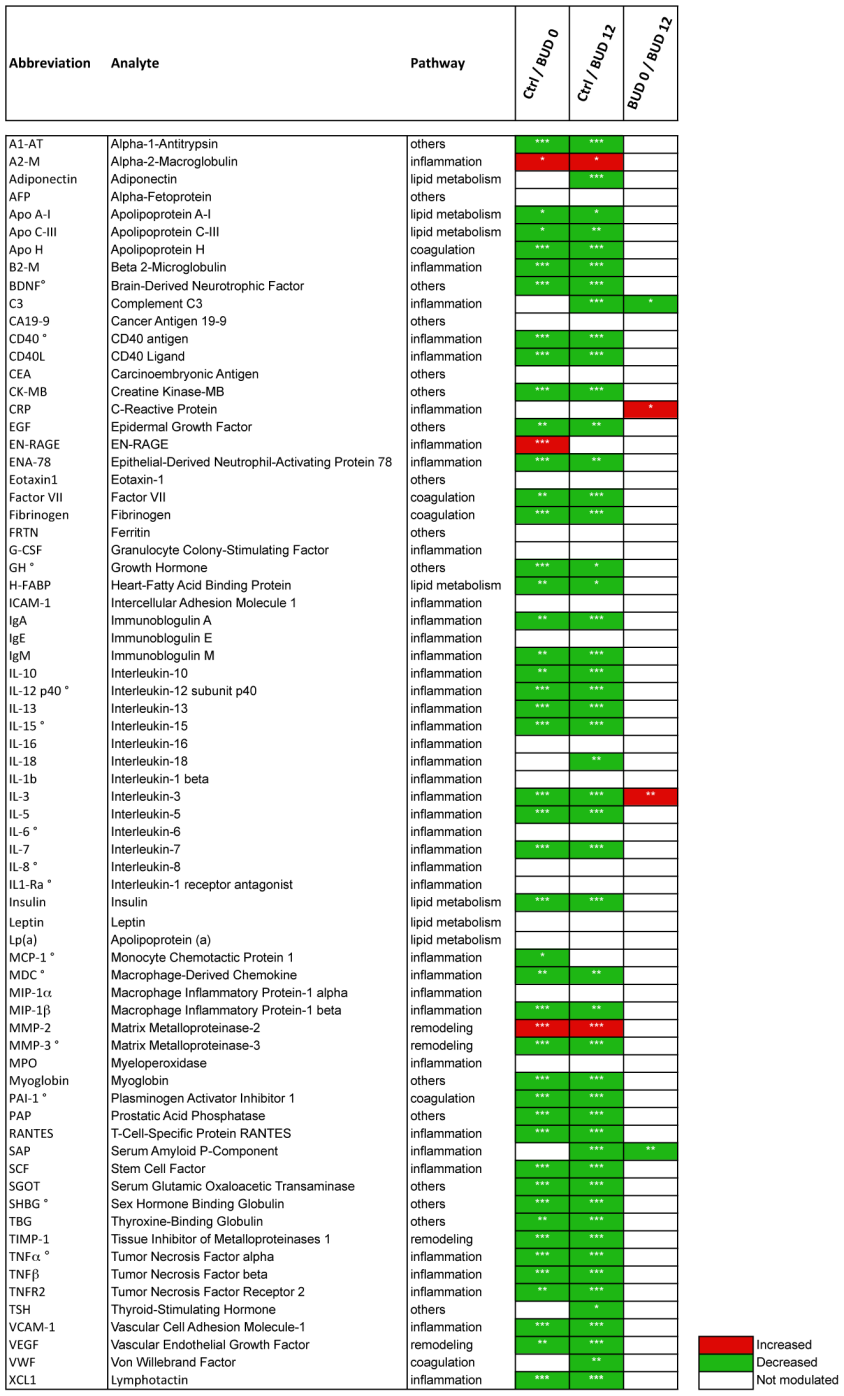
Univariate analysis of the measured serum proteins in healthy donors (Ctrl) and BUD patients at week 0 (BUD 0) or week 12 (BUD 12) p≤0.001 (***); p≤0.05 (**); p≤0.01 (*). The ° symbol shows the analytes associated with age, according to Spearman (Coefficient <−0.6).

Serum samples (100 µl) were sent on dry ice to Rules-Based Medicine Inc. (Austin, USA) for quantitative measurement of 88 proteins (Human Multi-Analyte Profile (MAP) v1.6). Details on the methodology used by this company can be found at www.rulesbasedmedicine.com. Briefly, samples were incubated with a mixture of fluorescently labeled microspheres, with each type conjugated to a different capture antibody. A mixture of biotinylated secondary antibodies was then added to label bead-captured antigen, then streptavidin-phycoerythrin added to the complex. Flow cytometry analysis with a Luminex 100 flow analyzer was used to quantify fluorescence signals for each antigen. Purified antigen standards were included to relate bead-based fluorescence to antigen concentration. A “Lowest Detectable Dose” (LDD) was also reported for each antigen that corresponds to mean fluorescence signal of 20 blanks +3 SD. The LDD is considered the lower limit at which the system can accurately calculate the concentration of an experimental sample, with confidence that the concentration is higher than that of a blank sample. We chose to keep only variables for which >50% measures were above the LDD in one of the control or patient group. This resulted in retention of 71 out of the 88 variables (Fig. 1). The lower assay limit (LAL) is each assay's working sensitivity as defined by the lowest concentration calibrator found on the standard curve that provides a quantifiable measurement above background. The LAL is typically lower than the LDD. Values below the LAL were replaced with a value that was 50% of the lowest value measured in the data set. Values above the maximum value of the standard curve were set at this maximum.

### Statistical analysis

Multivariate data analysis was performed with the SIMCA 13 software (Umetrics, Sweden) to predict clinical status based on protein signatures in 1) BUD patients before antibiotic treatment (BUD 0) versus healthy controls, 2) BUD patients 4 weeks after the end of the 8 week antibiotic treatment (BUD 12) versus healthy controls, 3) BUD 0 versus BUD 12 and 4) fast versus slow healers. We used conventional Principal Component Analysis (PCA) to gain a first insight into the general distribution of our dataset and detect outliers. In complement, Orthogonal Partial Least Squares-Discriminant Analysis (O-PLS-DA) was performed to identify the variables contributing to group classification [22]. The quality of each model was assessed by calculation of Fit (R^2^) and prediction ability (Q^2^) values. To further characterize univariate differences between controls, BUD 0 and BUD 12 patients, the non-parametric Wilcoxon ranksum (Mann-Whitney) test was used. Paired samples for BUD patients at week 0 and week 12 were compared using the Wilcoxon signed rank test. As this led to performing 71 tests for each comparison, we adjusted for multiple testing by defining as significant all p-values<0.05 that also guaranteed a false discovery rate (FDR) under 1% [23]. Univariate analysis and multiple testing adjustments were performed using R software (version 2.13.2, R Development Core Team, R Foundation for Statistical Computing, Vienna, Austria) and package QVALUE v1.0 [24]. The GraphPad Prism software (v5.0d, La Jolla, CA) was used for box-and-whisker plot representation, with outlier cut-off determined by Tukey's test. Outliers were kept in all statistical analyses and are represented by dots in box-and-whisker plots.

## Results

### Suppression of multiple serum proteins in BUD

**Figure 2:**
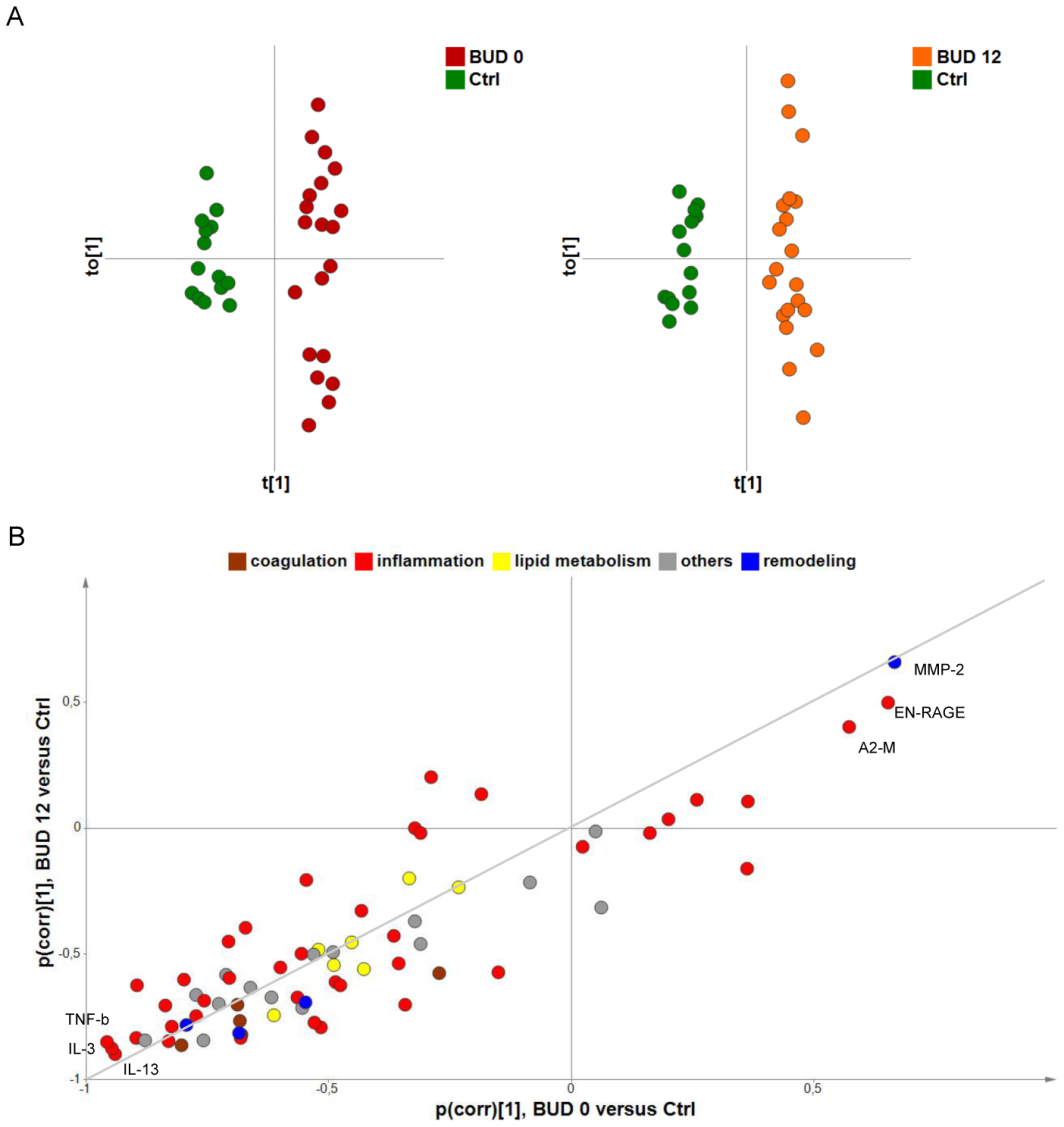
Suppression of multiple serum proteins in patients with BUD. A) O-PLS-DA of serum protein levels in healthy donors (Ctrl) versus BUD patients at week 0 (BUD 0, left) or week 12 (BUD 12, right). Score plots displaying predictive t[1] versus orthogonal t_O_[1] components are shown, with 95% confidence ellipses (based on Hotelling's T2 statistic) showing the absence of outliers. B) SUS-plot resulting from these O-PLS-DA models. Variables situated along the indicated diagonal are comparably modulated in BUD 0 and BUD 12, compared to Ctrl. The more reliable (pcorr[1] close to −1 and +1) are labelled.

Serum harvested from 20 patients at the time of BUD diagnosis (BUD 0), and 4 weeks after completion of antibiotic therapy (i.e. week 12, BUD 12) were compared to those of healthy controls from the same endemic community (Table 1). Samples were subjected to a multi-analyte profiling technology allowing for the quantitative and simultaneous determination of 88 proteins reflecting inflammation, lipid metabolism, coagulation, tissue remodelling and other pathways. Of the 88 targeted proteins, 71 met the inclusion criteria for further analysis (see Materials and Methods). PCA of the selected analytes resulted in a clear separation between BUD patients and healthy controls (R^2^ = 0.50; Q^2^ = 0.41), but not between patients at the beginning or end of antibiotic therapy (unshown data). O-PLS-DA improved the separation of controls versus BUD 0 (R^2^ = 0.96; Q^2^ = 0.92) and controls versus BUD 12 (R^2^ = 0.96; Q^2^ = 0.88) (Fig. 2A) but failed to better discriminate BUD 0 from BUD 12, suggesting that BUD induces alterations in serum proteins that are maintained after the end of antibiotic treatment.

Accordingly, all analytes distributed along the diagonal of a BUD 0/BUD 12 Shared and Unique Structures (SUS)-plot (Fig. 2B). Notably, a large proportion of the dots were concentrated in the lower left quadrant, indicating that BUD has a suppressive effect on most tested proteins, irrespective of their functional category. Univariate analyses confirmed that 44/71 serum proteins were down-modulated in BUD 0 compared to controls (Fig. 1). Because our cohorts of patients and controls did not match perfectly with regard to age, we verified potential links between variables and age a posteriori. This analysis identified 14 variables correlating negatively with age in controls, according to Spearman's test (Coefficient <−0.6) (Fig. 1 and Figure S1 in Text S1). Their down-regulation in patients may be due to an age bias and must be interpreted with caution. With regard to the variable with the highest correlation coefficient, MCP-1, we found that protein levels were not associated with age in BUD patients at Week 0 (Spearman coefficient R = 0.02). In a logistic regression model where BUD status was explained by MCP-1 levels at week 0, age was not significantly associated to disease and its introduction did not modify the relationship between BUD status and MCP-1 levels. Together, these findings thus indicated that the relationship between BUD and MCP-1 levels at week 0 was unlikely to be confounded by age. Notably, 43 of the 44 proteins that were down-regulated by BUD were still suppressed after 12 weeks, that is one month after completion of antibiotic therapy. Amongst the variations contributing the most to differentiate BUD patients from controls was the suppression of TNF-b, interleukin (IL)-3 and IL-13 and the induction of metalloproteinase MMP-2, damage associated molecule EN-RAGE (S100A12) and coagulation inhibitor A2-Macroglobulin (Fig. 1 and Fig. 2B).

### Broad but uneven down-modulation of inflammatory mediators in the peripheral blood of patients with BUD

**Figure 3:**
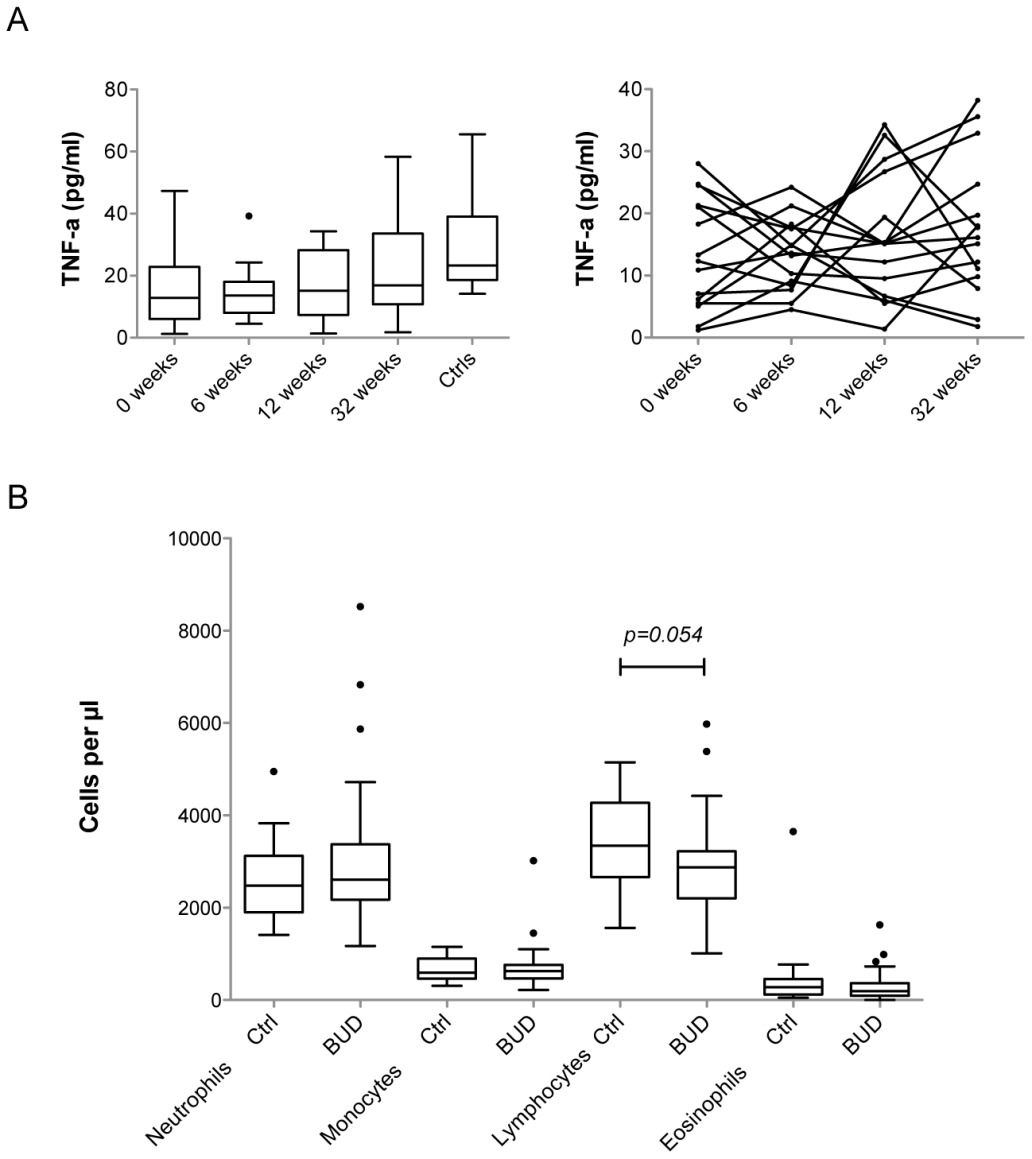
Down-modulated inflammatory mediators but normal leukocyte numbers in the peripheral blood of BUD patients. A) Evolution of the concentration of TNF-a in the serum of BUD patients, at the time of diagnosis (0 weeks) and 6, 12 and 32 weeks later, compared to healthy controls (Ctrls). The left panel shows the distribution of TNF-a concentrations at given time points as Box and Whiskers. The right panel shows the longitudinal variations of TNF-a in individual patients. (B) Leukocyte concentrations, presented as Box and Whiskers, in the blood of BUD patients and controls (Ctrl), as determined by complete blood counts.

The presence of an immunosuppressive signature in BUD, marked by the down-modulation of serum Macrophage inflammatory protein-1-beta (MIP-1b), Monocyte chemotactic protein-1 (MCP-1) and Interleukin (IL)-8 has been previously reported [20]. Although IL-8 was not significantly modulated in the present study, MIP-1b and MCP-1 were lowered in BUD 0, compared to controls (Figure S1 in Text S1). Furthermore, other inflammatory chemokines, such as the Regulated upon activation normal T cell expressed and secreted RANTES, the Macrophage-derived chemokine MDC and the Epithelial neutrophil-activating protein 78 (ENA-78), and a number of cytokines: IL-1a, IL-3, IL-5, IL-7, IL-10, IL-12_p40/p70_, IL-13, IL-15, IL-18, Tumor necrosis factor alpha and beta (TNF-a and -b), IL-10 and Stem cell factor SCF were reduced in patients (Figure S1 in Text S1). A significant decrease in the concentrations of soluble CD40, CD40 ligand (CD40L) and Beta-2-microglobulin (B2-M) was also observed, indicative of systemic defects in T cell activation. Longitudinal analyses of TNF-a and MCP-1 levels in serum at 0, 6, 12 and 32 weeks post diagnosis with the Luminex approach confirmed this general trend, in spite of important kinetic and inter-individual variations (as shown in Fig. 3A for TNF-a). Of note, BUD-associated defects affected the B cell compartment, as evidenced by the reduced levels of Immunoglobulin (Ig) A and IgM (Figure S1 in Text S1). However, IgE, IL-16 and Myeloperoxydase (MPO) were unchanged and EN-RAGE was up-regulated in patients, showing that the immunosuppressive effect of BUD is not uniform.

To determine if the down-modulation of lymphocyte-derived cytokines, chemokines and Igs in BUD patients could be due to infection-induced cell death, we recruited 10 additional patients and healthy controls (Table 3) and assessed CD4^+^, CD8^+^ and CD19^+^ peripheral blood lymphocytes by means of complete blood count and fluorescence activated cell sorting (FACS). Since CD62-L expression was shown to decrease in T cells upon exposure to mycolactone [17], this homing molecule was also included in the staining panel. No significant difference in the mean expression of CD4, CD8 or CD62-L by T cells nor in the relative abundance of lymphocytes expressing these receptors could be demonstrated in BUD patients (Figure S2 in Text S1). A minor increase in the mean expression of CD19 in B cells was observed in patients, which might be due to the presence of two outliers. While one would expect variations in the numbers of white blood cells in infected individuals, absolute counts of neutrophils, monocytes and eosinophils were comparable in BUD patients and controls (Fig. 3B). The total number of lymphocytes was slightly reduced in patients, but remained within normal values (1000–3700 cells per µl). In conclusion, BUD alters durably the circulating levels of a large array of inflammatory mediators without perturbing the composition of peripheral blood leukocyte populations.

### Abnormal metabolic responses to *M. ulcerans* infection

Infection with pathogenic microorganisms typically induces the production of pro-inflammatory cytokines by innate immune cells, which in turn triggers a reprogramming of protein synthesis in the liver. This so-called “acute phase reaction” (APR) is thought to result primarily from the activation of hepatic receptors by TNF-a, IL-1 and IL-6, and entails the active production and release in the peripheral circulation of proteins trapping micro-organisms, activating complement and modulating the host's immune response. Since IL-1b and IL-6 were unchanged and TNF-a was suppressed in patients, we examined the impact of the disease on acute phase reactants. In spite of the presence of progressive lesions in their skin, BUD patients at Week 0 (BUD 0) and controls had equivalent mean levels of C-reactive protein (CRP) and Complement Factor 3 (C3), while Fibrinogen, Plasminogen-activator inhibitor type 1 (PAI-1), Serum amyloid P-component (SAP), and Alpha-1 antitrypsin (A1-AT) were in fact suppressed by infection (Fig. 1 and Figure S1 in Text S1). The proteinase inhibitor alpha-2 macroglobulin (A2-M) was the only exception to this rule.

**Figure 4:**
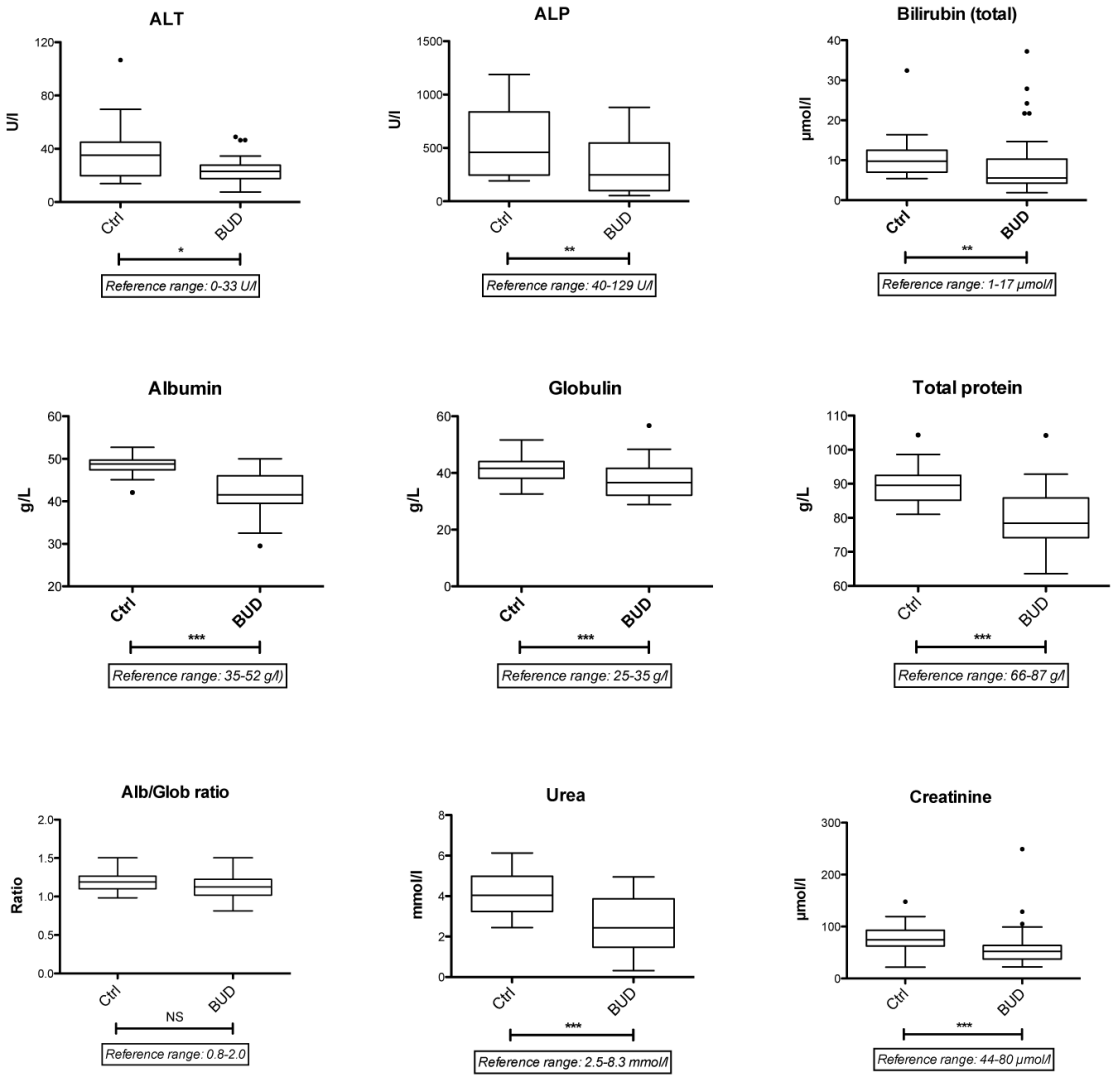
Abnormal metabolic responses to *M. ulcerans* infection. Effect of BUD on the serum level of various indicators of liver and renal function is shown. Data are presented as Box and Whiskers in Ctrl and BUD patients. *p<0.01, **p<0.005, ***p<0.001, NS: not significant.

To investigate the possibility that liver functionality was altered in patients, we conducted a series of biochemical assays in the serum of patients and controls from Cohort 2. ALT, ALP and bilirubin (both total, direct and indirect) were down-regulated in patients while GGT were unchanged (Fig. 4 and unshown data), ruling out the possibility that BUD causes liver damage. Albumin is exclusively produced by the liver and negatively regulated during APR. Surprisingly in the absence of positive acute phase reactants, the serum levels of albumin were reduced in patients. Consistent with the down-modulation of alpha-, beta- and gamma-globulins (A1-AT, B2-M and Igs), BUD patients also had reduced globulin levels, compared to controls. As a result of the decrease in both albumin and globulins, patients displayed significantly lower levels of total serum proteins. However their mean concentrations of albumin, globulin, total proteins and albumin/globulin ratios remained within reference range. Blood urea and creatinine were diminished in patients, while the blood urea nitrogen/creatinine ratio remained normal, arguing against acute kidney failure or dehydration problems (Fig. 4). Together, these data suggested that BUD impacts on the host's metabolism of protein, without damaging liver, kidney or muscles.

### Alterations in blood lipid transporters and free fatty acids

**Figure 5:**
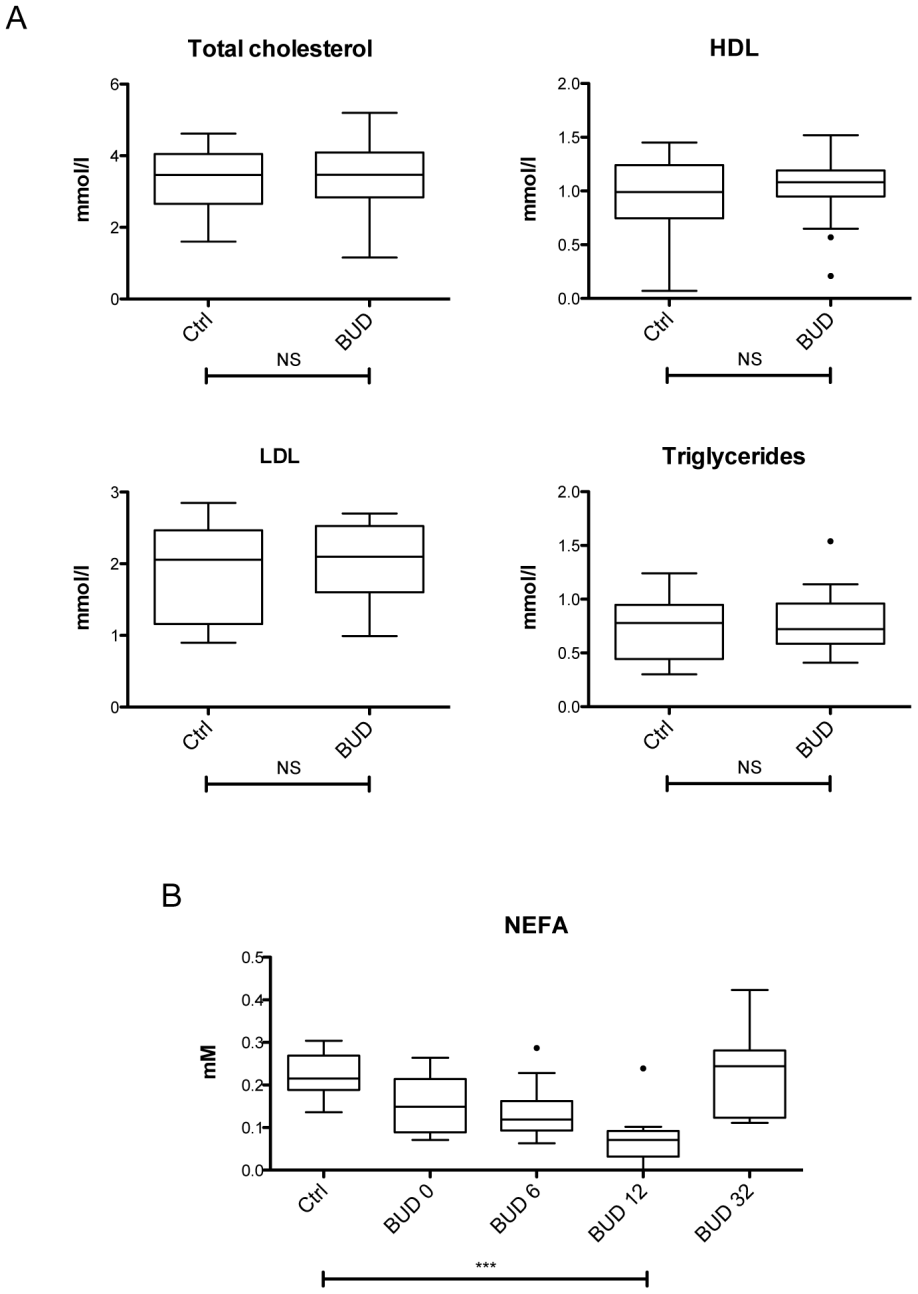
Decreased levels of free fatty acids in the peripheral blood of patients with BUD. A) Concentration of blood lipids in patients and controls (Ctrl). B) Serum levels of NEFA in patients at week 0, 6 12 or 32, and in controls (Ctrl). Data are presented as Box and Whiskers, ***p<0.001, NS: not significant.

Our screen also identified perturbations in proteins that are not involved in immune responses and APR, such as regulators of lipid metabolism and transport. Apolipoprotein (Apo) A-I, Apo C-III, Apo H, Fatty acid binding protein (FABP) and Insulin were suppressed by 50% on average in BUD patients, while Lipoprotein (Lp) was unchanged (Fig. 1 and Figure S1 in Text S1). To evaluate the possible impact of these changes on lipid generation and transport, we assessed blood lipids and lipoproteins in patients and controls (Tables 2 and 4). Total cholesterol, HDL, LDL and triglycerides were unaffected by the disease (Fig. 5A). In contrast, the serum level of non-esterified fatty acids (NEFA) was significantly reduced in BUD patients, with minimal values detected 3 months post diagnosis and restoring after 8 months (Fig. 5B). Since NEFAs are water-insoluble and transported in the circulatory system bound to albumin, their lower serum concentration may reflect the reduced availability of their transporter.

### Modulation of tissue remodelling markers

Consistent with the destruction of cutaneous and subcutaneous tissues by *M. ulcerans*, a number of markers of tissue remodelling were altered in patients. The circulating concentrations of matrix metalloproteinase-3 (MMP-3) and vascular endothelial growth factor (VEGF), which are both crucial for connective tissue remodelling and wound repair, were stably down-regulated by BUD (Fig. 1 and Figure S1 in Text S1). However MMP-2, which also contributes to regulation of vascularisation and inflammation, was modulated in the opposite direction. EN-RAGE, another important regulator of vascular remodelling, was also up-regulated by *M. ulcerans* infection.

### Differential inflammatory and metabolic profiles in faster healing patients

**Figure 6:**
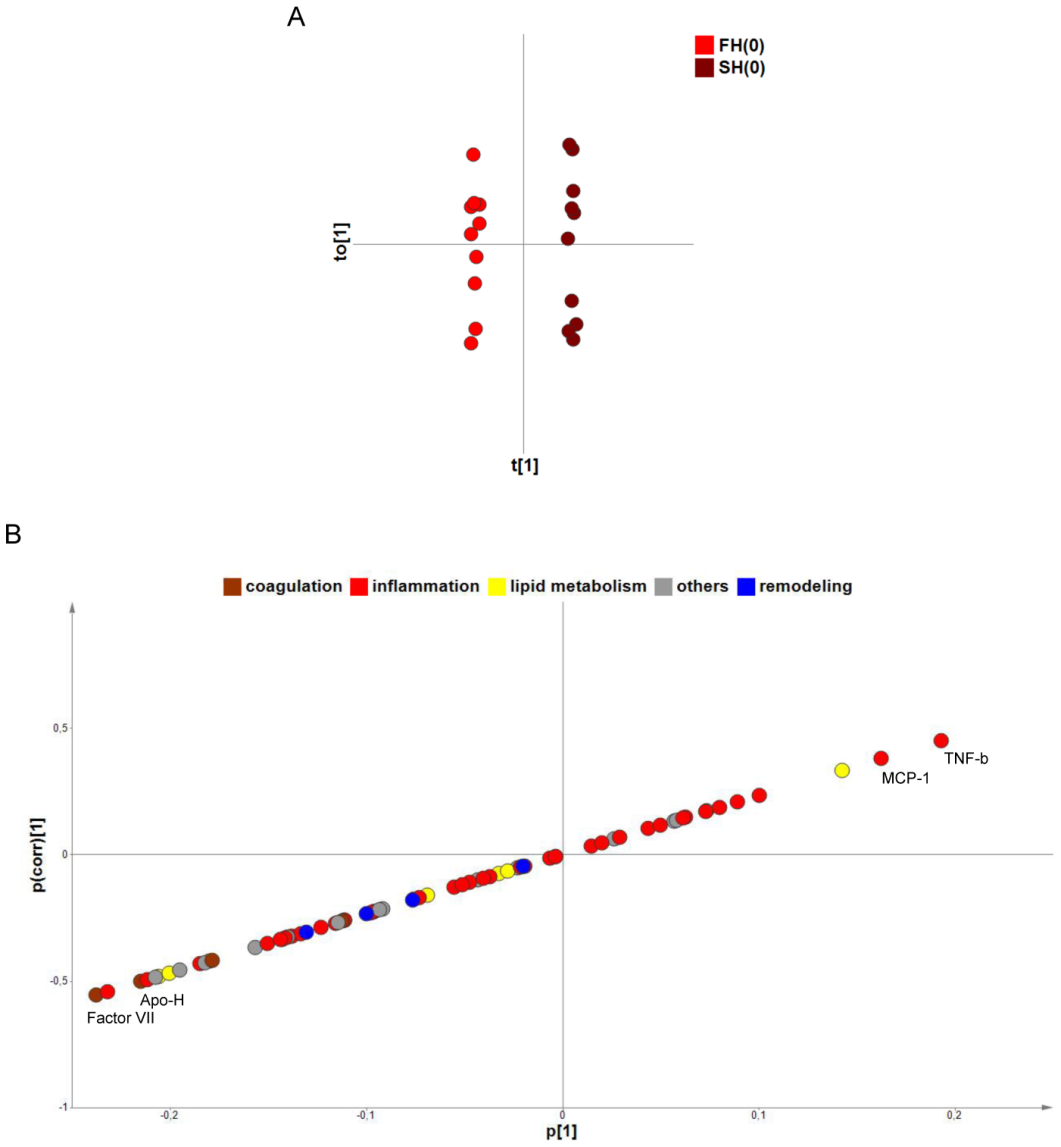
Differential protein signature in fast and slow healers. A) O-PLS-DA of serum protein levels in fast and slow healers, before the beginning of antibiotic therapy. The score plot for this model (predictive t[1] versus orthogonal t_O_[1]) with a 95% confidence ellipse are shown. B) S-plot illustrating the modelled covariance (p[1]-axis) and correlation (pcorr[1]-axis) of variables in FH and SH. The p[1]-axis describes the magnitude of each variable, while the pcorr[1]-axis represents the reliability of each variable (between +/−1). The most discriminating variables, at both extremities of the X-Y diagonal, are labelled.

To identify biological correlates of healing, we then subjected our data to a multivariate analysis comparing serum protein levels measured at the time of inclusion in antibiotic therapy in “fast healers” (FH) and “slow healers” (SH). For this purpose, a cut-off healing time of 12 weeks was chosen, as it divided the BUD cohort into two sub-groups of 10 patients that were comparable in terms of gender ratio and category of lesion (Table 1). O-PLS-DA of serum proteins resulted in a clear separation between FH and SH (R^2^ = 1.00; Q^2^ = 0.43) (Fig. 6A), but not between FH or SH between week 0 and week 12 (unshown data). Compared to FH, SH displayed relatively higher initial levels of TNF-b and MCP-1; and lower levels of Factor VII and Apo H (Fig. 6B). Although these initial differences between fast and slow healers must be confirmed with larger cohorts of patients, the data in Figure 6 suggest that impaired healing is associated with distinct inflammatory and coagulation defects.

## Discussion

Our multi-analyte profiling of the serum of BUD patients revealed the suppression of a large array of circulating proteins. The analytes targeted by our study were representative of the major categories of serum proteins, and their global suppression reflected by a 10% decrease in total protein. Since some proteins were up-regulated by the disease, the down-regulatory effect of BUD is unlikely to be due to plasma volume expansion. The absence of obvious swelling in patients, and the fact that their mean weight, height, BMI, heart rate and blood pressure were comparable to those of controls (Table S1 in Text S1, Cohort 2) also argue against this possibility. Invasion of pathogenic microorganisms or tissue injury typically induce a localized inflammation triggering the production of cytokines, chemokines, immunoglobulins by the immune system and acute phase proteins by the liver. This is exemplified by the potent pro-inflammatory cytokine and acute phase responses that follow infection with *M. tuberculosis*, and their reduction upon antibiotic treatment [25]. Severe burns cause acute inflammation and production of CRP, accompanied with extensive muscle catabolism and protein anabolism in the wound [26], [27]. In spite of the presence of proliferating bacilli and necrotic material in Buruli ulcers, we found no evidence of such inflammatory responses and acute phase reaction in patients, suggesting that *M. ulcerans* successfully evades immune recognition. To our knowledge, the combined suppression of inflammatory mediators and major serum proteins is a unique feature of *M. ulcerans* infection.

We previously reported that BUD patients display a distinctive signature of chemokine suppression in their peripheral blood [20]. Here, we validate this observation in independent cohorts of patients and endemic controls, and extend the list of inflammatory mediators that are down-regulated by *M. ulcerans* infection. BUD patients showed a broad inhibition of the circulating levels of multiple cytokines, chemokines and Igs. In contrast, the granulocyte-derived EN-RAGE was induced in patients with BU and down-modulated in 14/20 patients undergoing treatment. As previously reported for a number of inflammatory disorders, this member of the S100 family of calcium-binding proteins may be of clinical value in the assessment of BUD [28]. With the exception of EN-RAGE, our data support the lack of inflammation surrounding necrotic lesions [4] and the functional defects of circulating T lymphocytes in BUD patients [20], [29]. The concentration and viability of monocytes, neutrophils, T and B cell populations were intact in the peripheral blood of BUD patients, showing that *M. ulcerans* infection does not impair innate and acquired immune responses by depleting these immune cell subsets.

Importantly, BUD patients do not show increased susceptibility to microbial infections [2]. In the mouse model, the control of *Listeria monocytogenes* infection is not altered by co-infection with *M. ulcerans* [30]. The immunosuppressive signature in the peripheral blood of patients with BUD is therefore not indicative of an immunodeficient status. Our longitudinal analysis of individual patients with BUD showed that down-regulation of TNF-a is incomplete and fluctuant during treatment (Fig. 3A), suggesting that inflammation is tuned down by infection but not abolished. Intriguingly, immune modulation persisted for weeks after the elimination of *M. ulcerans* bacilli with antibiotic treatment. Structurally intact mycolactone has been found in significant amounts in ulcer exudates after completion of antibiotic therapy [31]. Since mycolactone diffuses from infected skin into the peripheral blood and accumulates in internal organs such as spleen and liver [32], the long-lasting down-regulation of inflammatory markers in the peripheral blood of treated patients may thus reflect the slow release of this compound into the circulation. Recent progresses in the techniques allowing mycolactone extraction and quantification from peripheral blood and tissues may help address this hypothesis [33], [34].

Inflammation is highly connected to the biology of adipose tissues [35]. At the tissue level, this is illustrated by the localized infiltration of inflammatory cells into the fat tissues of obese subjects. At the systemic level, obesity is associated with elevated pro-inflammatory cytokines TNF-a and IL-6, NEFA and basal concentrations of insulin. Interestingly, BUD patients had an opposite phenotype, with lower TNF-a, insulin and NEFAs. Adipocytes produce a vast array of signalling proteins including VEGF, TNF-a, Adiponectin, A2M, VCAM-1, CRP, PA1, MCP-1, RANTES and TIMPs, all of which were down-modulated by the disease. It will be interesting to determine if these alterations are the direct consequences of a selective inhibition of protein synthesis by mycolactone in adipocytes, as previously shown in monocytes [19]. Alternatively, as BUD affects the circulating levels of proteins produced by various tissues including the liver and muscle tissues, they may reflect a central regulation of the host's metabolism via hormones.

Protein requirement increases during wound healing, and malnutrition is a risk factor for pressure ulcers. As a negative acute phase reactant that is affected by inflammation, albumin typically correlates inversely with infection, trauma, surgery, burns, or oedema. Decreased levels of serum albumin is considered an indicator of morbidity and mortality, while increased levels reflect improvement in clinical status [36]. In the present work, we observed a significant reduction in the mean serum albumin and total protein levels of BUD patients. Although none of the individuals recruited in this study showed severe signs of malnutrition, 42–50% of patients and controls were mildly to severely thin, according to WHO criteria. Since these proportions were comparable in patients and controls, it is unlikely that poor nutritional status is a risk factor for BUD. However, research into the nutritional requirements of patients with pressure ulcers suggests that it may impair BU healing. Based on our results, it would be useful to determine if *M. ulcerans* infection impairs body protein anabolism.

Our comparison of good vs. bad responders suggested that slowly healing patients have a differential profile of alterations in the inflammatory and coagulation pathways at the start of antibiotic therapy. Since the proportion of pre-ulcerative and ulcerative forms at inclusion was comparable in fast and slow healers, and both groups had comparable mean diameter of lesion (Table S1 in Text S1, Cohort 1), these differences cannot be explained by differential pathogenesis. Whether differences in the inflammatory and coagulation pathways exist before infection and predispose to *M. ulcerans* infection will require further work. Yet, our observation that cytokines and chemokines that are down-regulated by BUD slowly recover normal values (Fig. 2A and our previous findings [20]) argues against this hypothesis. Since their return to normal level does not coincide with the end of antibiotic treatment nor ulcer resolution, we propose that alterations is serum proteins of treated patients reflect the persistence of mycolactone or the long-lasting effects of infection-induced perturbations in the host's metabolism.

## Supporting Information

Text S1 *Table S1:* Information on each individual recruited in this study. *Figure S1:* Modulation of serum proteins by BUD. Data obtained for each of the 71 analytes are presented as Box and Whiskers in healthy controls (Ctrl) and patients at the beginning of antibiotic therapy (BUD 0) or 4 weeks after completion of treatment (BUD 12). *Figure S2:* FACS analysis of B and T cell populations in the peripheral blood of patients with BUD, compared to healthy controls (Ctrl). Data are percentage of lymphocytes (as gated on FSC/SSC) and mean fluorescence intensities (MFI) for CD4, CD8, CD19 and CD62-L positive cells, presented as Box and Whiskers *p<0.01, **p<0.005, ***p<0.001, NS: not significant.
(PPT)
