# Supplementary figures and images for: Combined Inflammatory and Metabolic Defects Reflected by Reduced Serum Protein Levels in Patients with Buruli Ulcer Disease
Source: PLoS Negl Trop Dis. 2014 Apr 10;8(4):e2786. doi: 10.1371/journal.pntd.0002786 (PMC3983110; doi:10.1371/journal.pntd.0002786)

## Slide 1
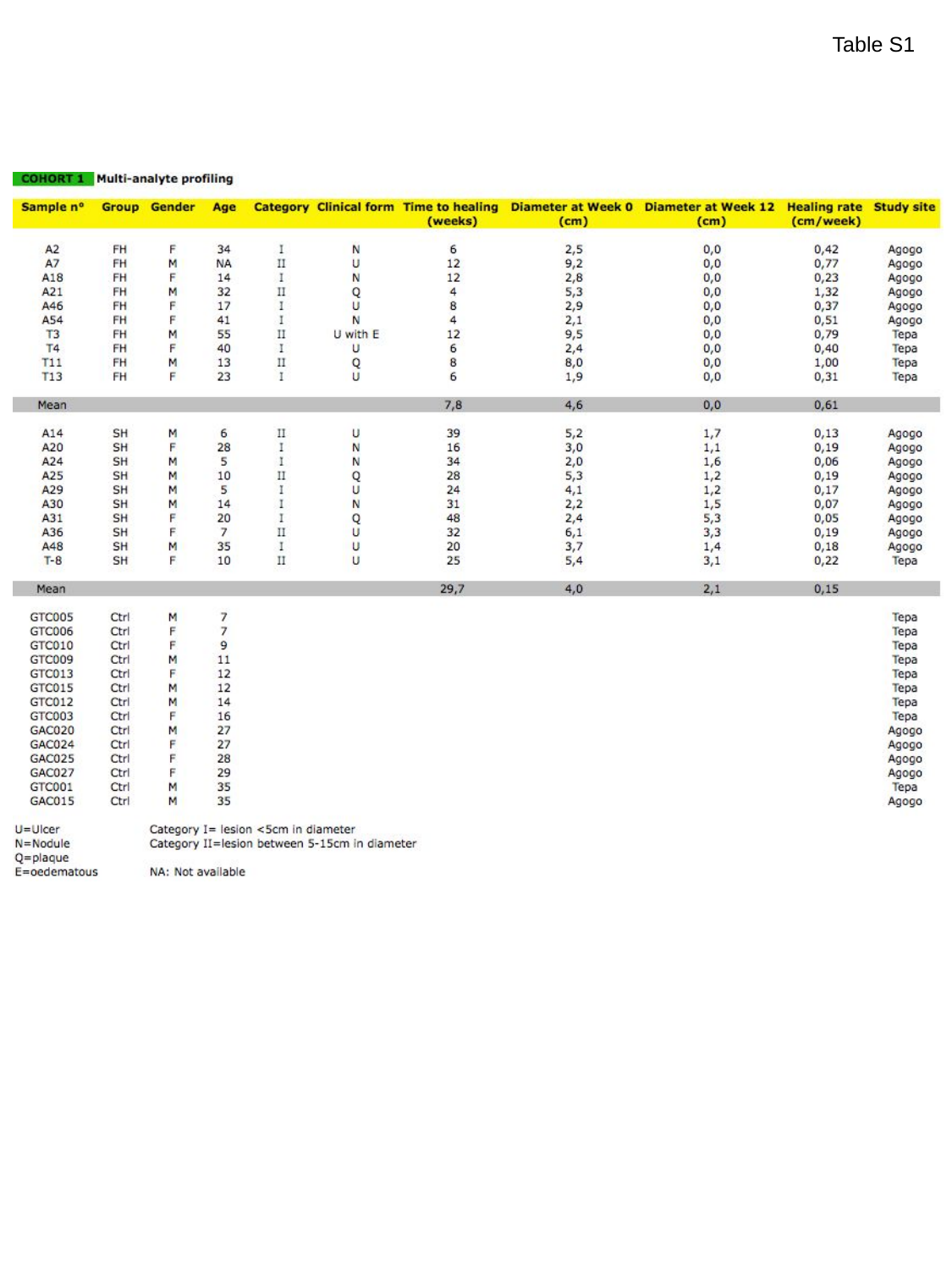

Table S1

## Slide 2
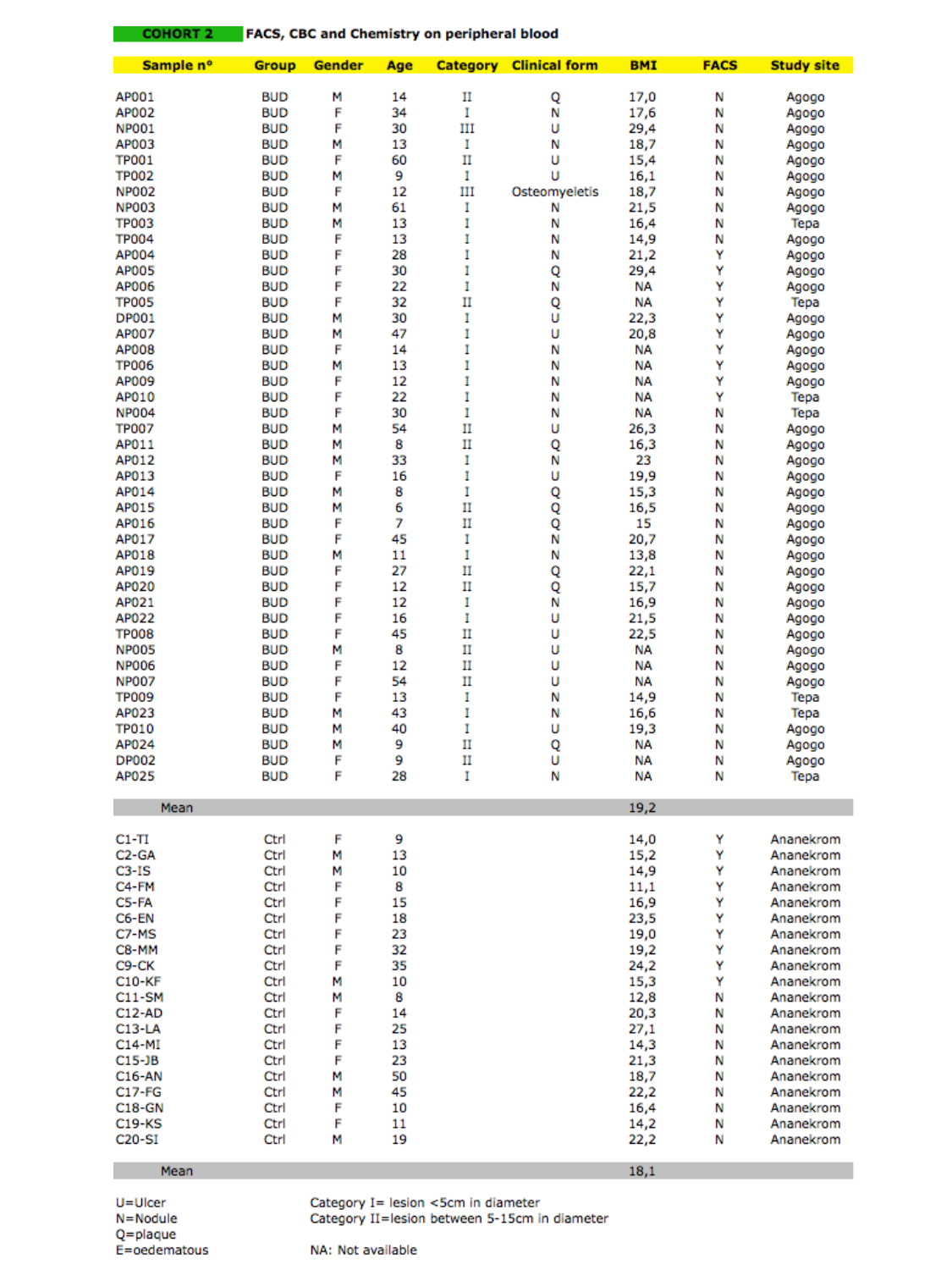

## Slide 3
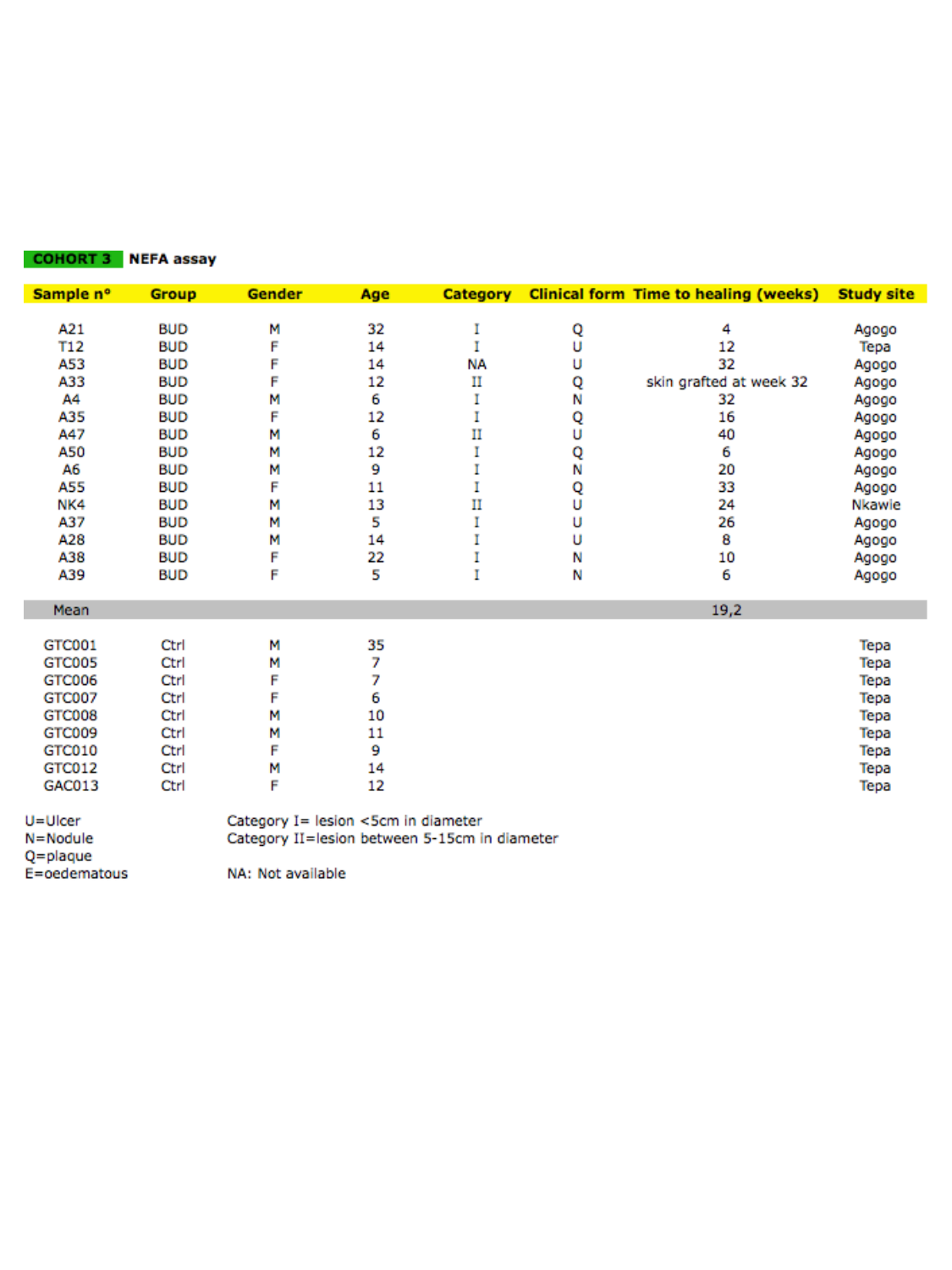

## Slide 4
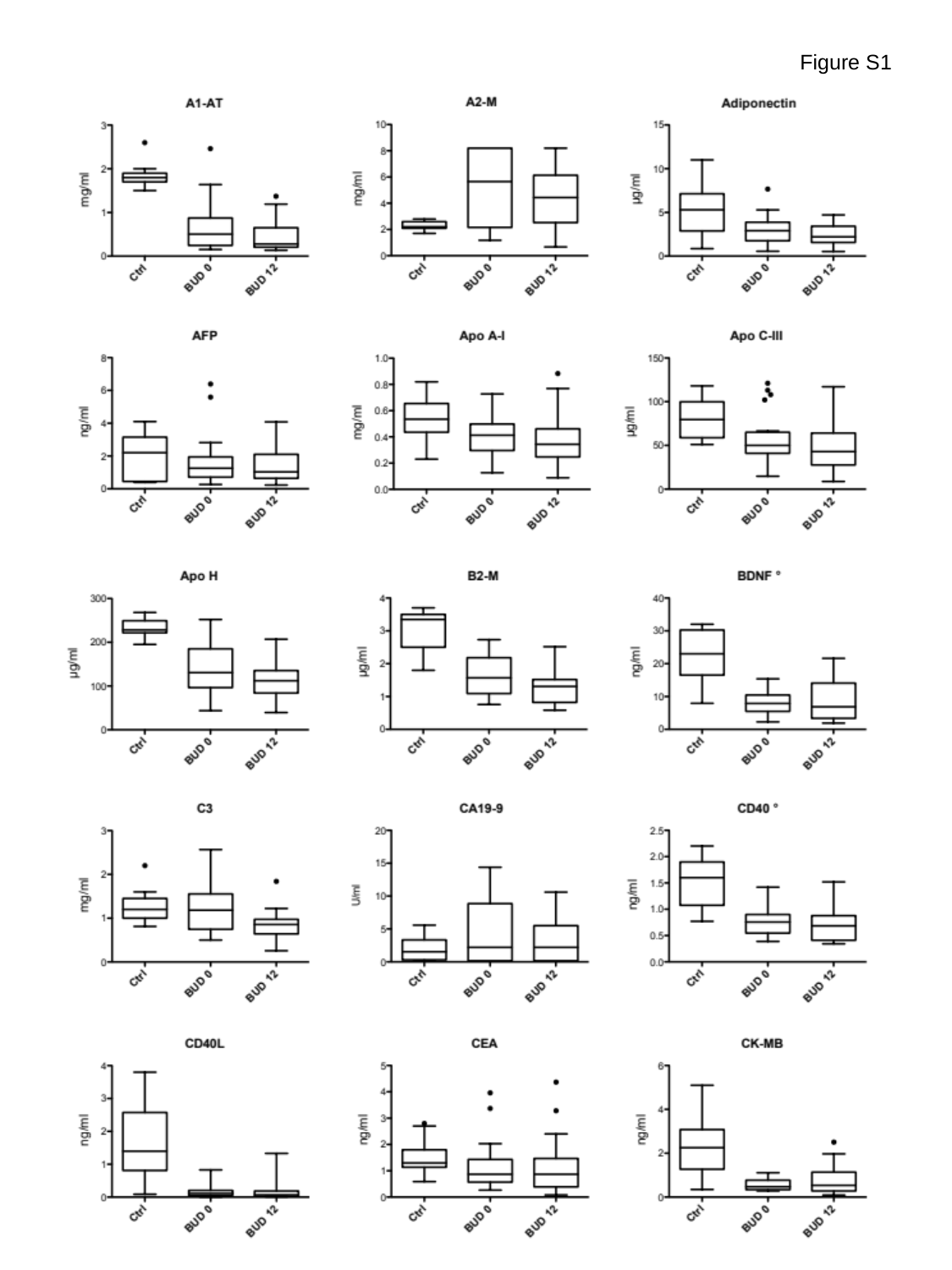

Figure S1

## Slide 5
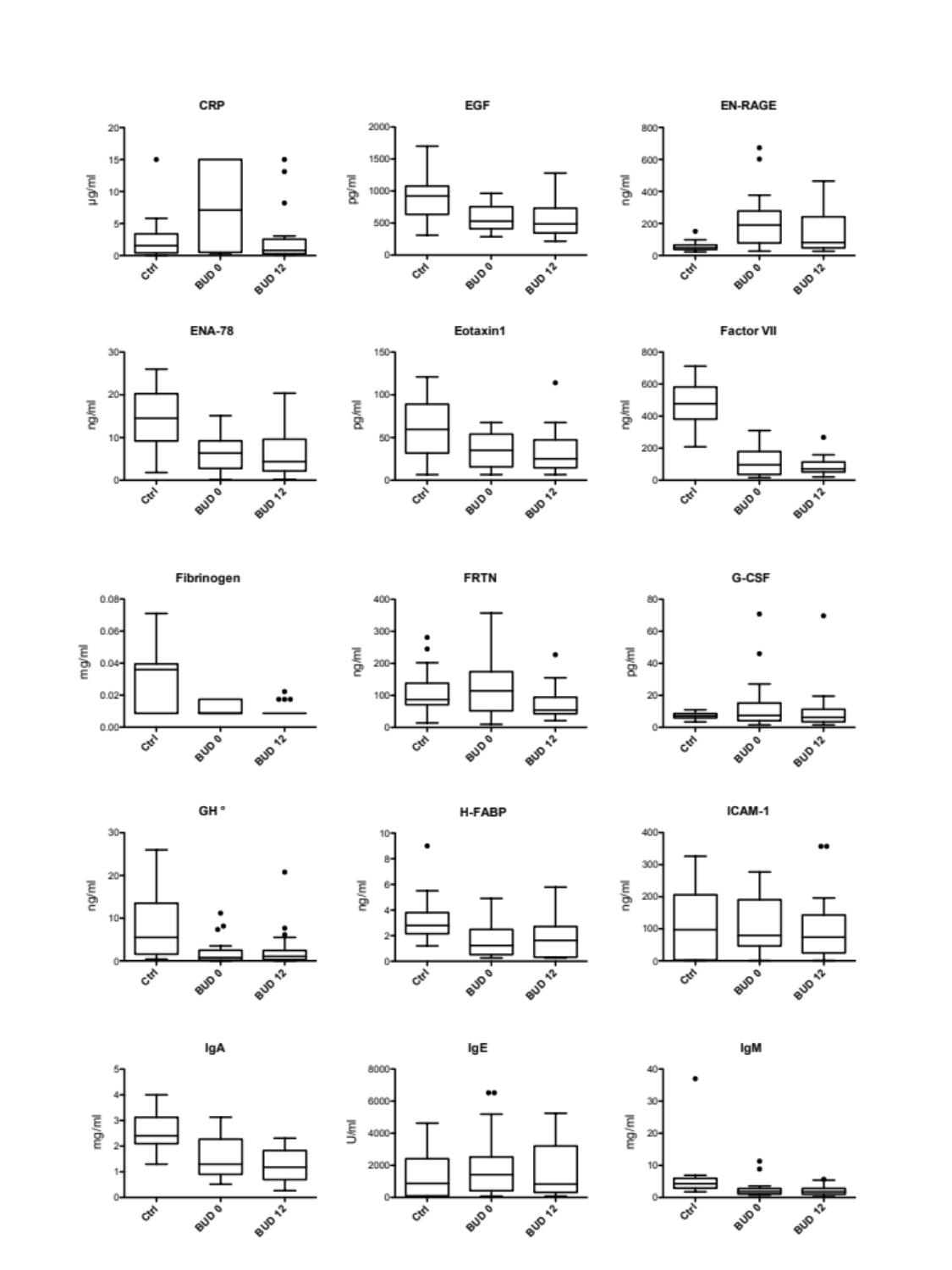

## Slide 6
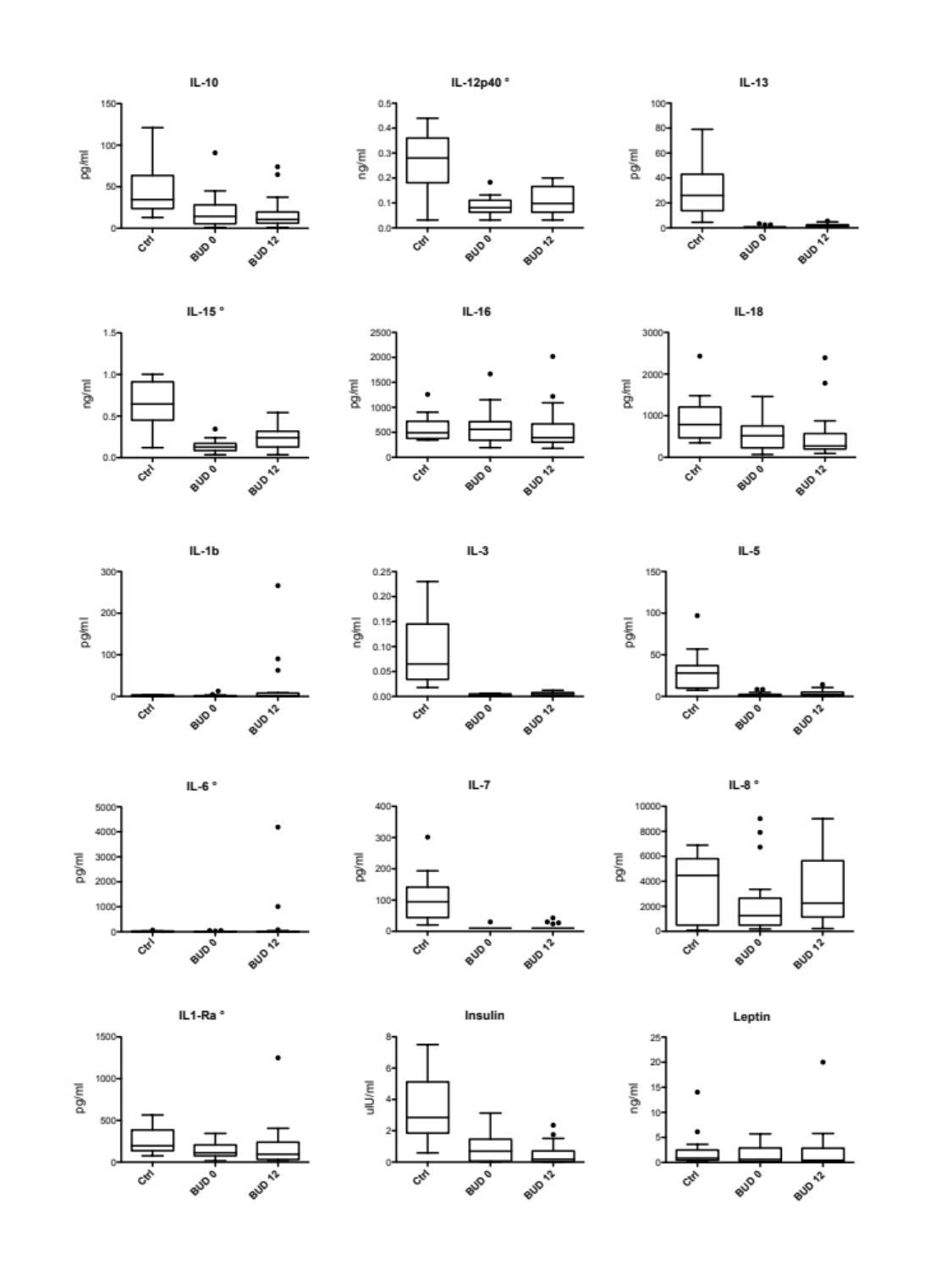

## Slide 7
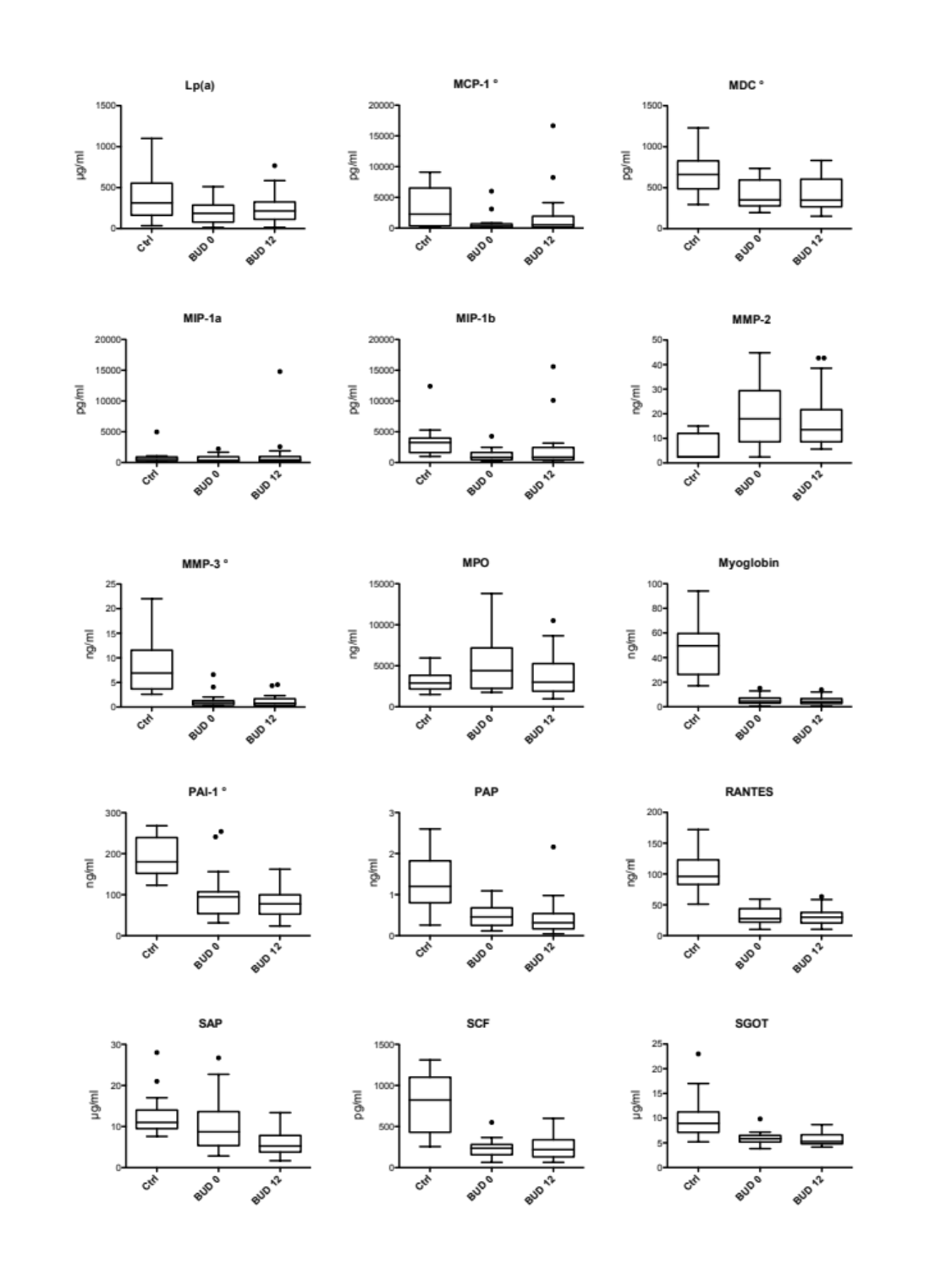

## Slide 8
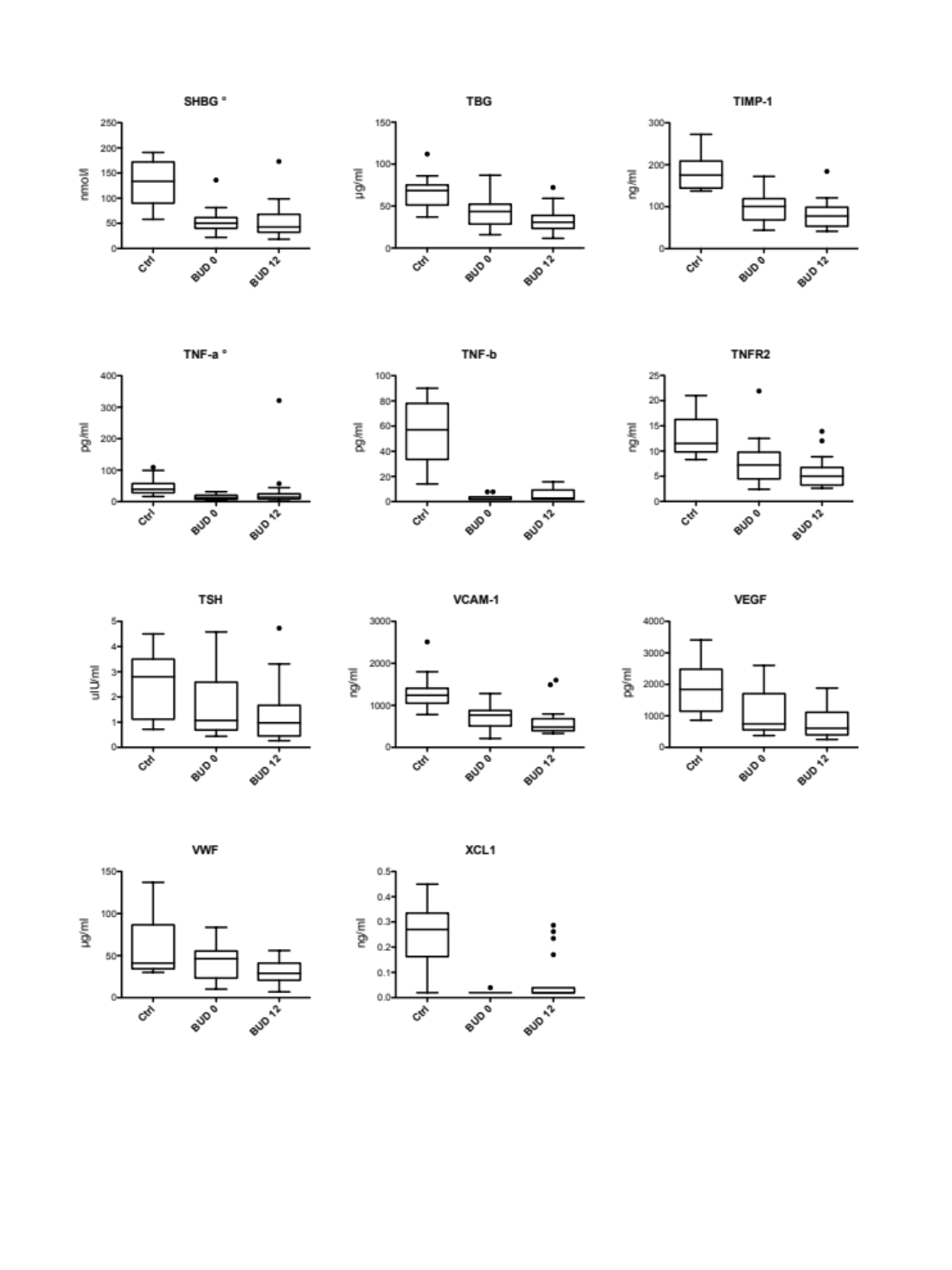

## Slide 9
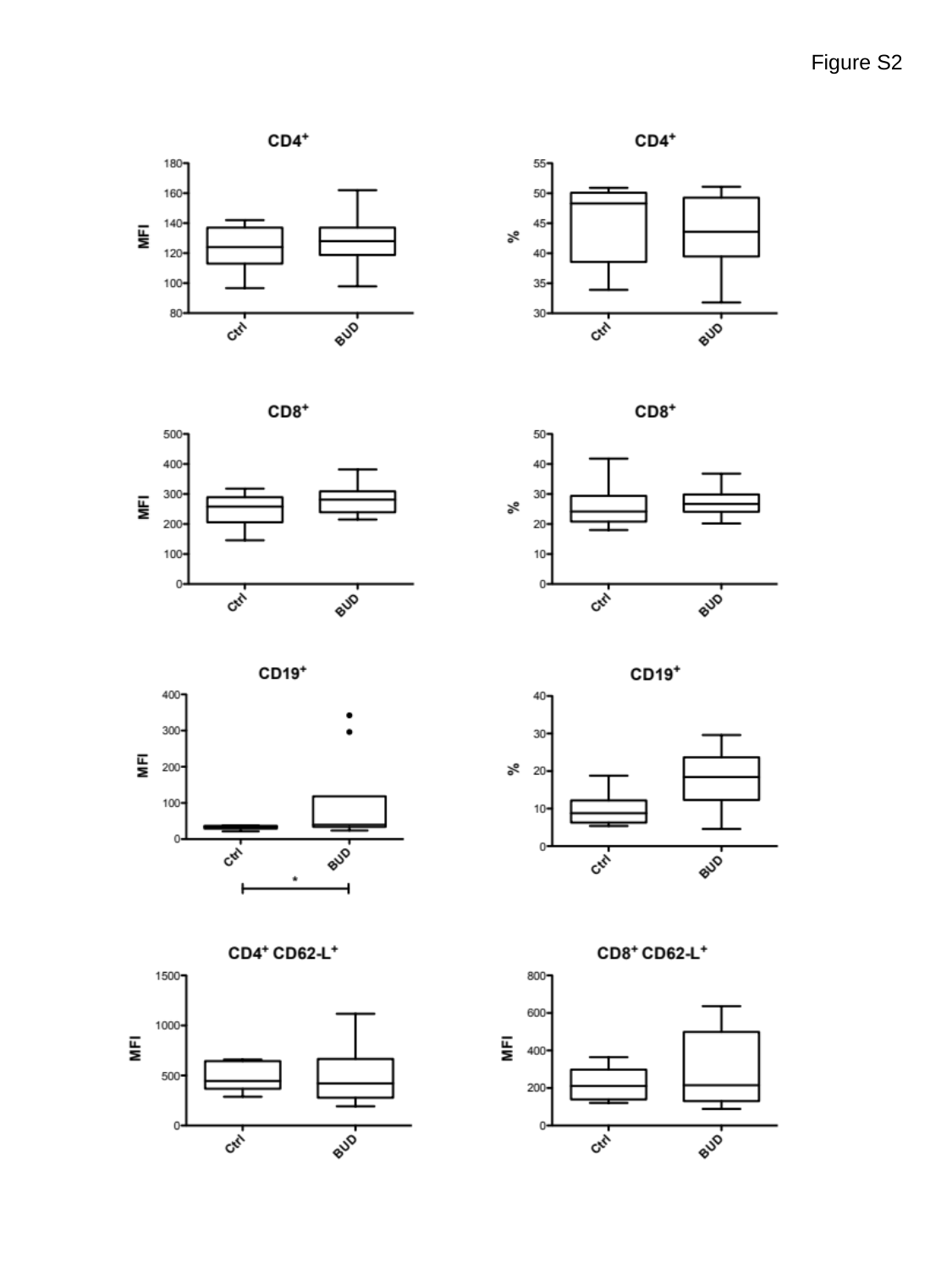

Figure S2

Supplement: Text S1 — Table S1: Information on each individual recruited in this study. Figure S1: Modulation of serum proteins by BUD. Data obtained for each of the 71 analytes are presented as Box and Whiskers in healthy controls (Ctrl) and patients at the beginning of antibiotic therapy (BUD 0) or 4 weeks after completion of treatment (BUD 12). Figure S2: FACS analysis of B and T cell populations in the peripheral blood of patients with BUD, compared to healthy controls (Ctrl). Data are percentage of lymphocytes (as gated on FSC/SSC) and mean fluorescence intensities (MFI) for CD4, CD8, CD19 and CD62-L positive cells, presented as Box and Whiskers *p<0.01, **p<0.005, ***p<0.001, NS: not significant. (PPT) [file pntd.0002786.s001.ppt]
